# Mechanisms of emerging technologies for inactivating foodborne viruses

**DOI:** 10.1128/aem.00242-25

**Published:** 2025-08-19

**Authors:** Ruthchelly Tavares da Silva, Yago Alves de Aguiar Bernardo, Louise Iara Gomes de Oliveira, Carlos Adam Conte-Junior, Marciane Magnani

**Affiliations:** 1 Laboratory of Microbial Processes in Foods, Department of Food Engineering, Technology Center, Federal University of Paraíba 734210 , João Pessoa, Paraíba, Brazil; 2 Laboratory of Advanced Analysis in Biochemistry and Molecular Biology (LAABBM), Department of Biochemistry, Federal University of Rio de Janeiro (UFRJ), Cidade Universitária, Rio de Janeiro, Brazil; 3 Center for Food Analysis (NAL), Technological Development Support Laboratory (LADETEC), Federal University of Rio de Janeiro (UFRJ), Cidade Universitária 28125, Rio de Janeiro, Brazil; 4 Analytical and Molecular Laboratorial Center (CLAn), Institute of Chemistry (IQ), Federal University of Rio de Janeiro (UFRJ), Cidade Universitária 28125, Rio de Janeiro, Brazil; 5 Graduate Program in Food Science (PPGCAL), Institute of Chemistry (IQ), Federal University of Rio de Janeiro (UFRJ), Cidade Universitária 28125, Rio de Janeiro, Brazil; Anses, Maisons-Alfort Laboratory for Food Safety, Maisons-Alfort, France

**Keywords:** foodborne virus, non-thermal processing, virus surrogates, viral contamination, virus inactivation

## Abstract

Contamination of food and water by viruses is a major public health issue worldwide. Several viruses are associated with foodborne outbreaks, with norovirus and hepatitis A virus being the primary causes of foodborne outbreaks, followed by hepatitis E virus and rotavirus. These viruses are responsible for the majority of outbreaks reported globally, representing a significant challenge to food safety; therefore, effective viral inactivation processes are needed. This review presents and discusses recent research involving emerging technologies used for the inactivation of foodborne viruses, emphasizing the mechanisms involved in the process, their effectiveness, and the main challenges associated with the application. Traditional methods, like heat treatments (pasteurization) and sanitizers (organic acids), are effective but have drawbacks, such as the nutritional and sensory losses of food. Novel technologies to overcome the limitations of thermal treatments and guarantee food safety have been developed, such as UV C light (UV-C), cold plasma, high-pressure processing (HPP), and ultrasound. These methods have been shown to be effective in inactivating viruses in fresh foods (fruits, vegetables, and seafood), beverages, and food-contact surfaces, without compromising food properties. Among these technologies, HPP and UV-C were the most studied. HPP compromises the structural integrity of the virus, while UV-C induces photochemical damage to viral DNA and RNA. These alterations, combined with other physical and chemical effects, contribute to the destruction of viral genetic material, leading to viral inactivation. Despite their effectiveness, non-thermal technologies still face barriers, such as strict regulations and high costs, which limit their widespread application.

## INTRODUCTION

Access to sufficient, safe, and nutritious food is an important component of public health management, and foodborne diseases have profound implications for human health and life (1). According to the World Health Organization (WHO), over 200 diseases are caused by eating food contaminated with microbial agents or chemical substances. Furthermore, 1 in 10 people become ill after eating contaminated food (2). In the Americas alone, 77 million people fall ill and more than 9,000 people die every year from foodborne illnesses (3). In addition to morbidity and mortality, diseases caused by enteric viruses impose a severe economic burden (4).

Among microbiological agents posing a risk to human health, foodborne viruses have gained increasing importance in recent decades (5). Human norovirus (HuNoV) and hepatitis A virus (HAV) stand out due to the large number of reported outbreaks (6). HuNov, for example, causes up to 685 million cases per year and 200,000 deaths, including 200 million cases among children under 5 years of age (7). Other viruses, such as the hepatitis E virus (HEV) and rotavirus (RV), are also associated with foodborne diseases (8, 9).

Unlike bacteria, viruses require a host to survive, as they have a simple structure, with a nucleocapsid formed by DNA or RNA as genetic material protected by proteins. Therefore, viruses cannot replicate in foods that may serve as a vehicle for infection (10, 11). Enteric viruses can survive in foods, water, and food processing environments (12). Food contamination by viruses can occur at any point in the food chain, mainly through the use of water contaminated with fecal matter or poor hygienic conditions of workers, and transmission to humans occurs via the fecal-oral route, person-to-person contact, or consumption of contaminated food or water (11, 13). Outbreaks of enteric viruses are frequently associated with the consumption of contaminated fresh produce, such as leafy greens, frozen and fresh fruits, bulb vegetables, and shellfish (14).

Due to the ability of enteric viruses to persist in various food products and their role in viral foodborne outbreaks, increasing attention has been given to methods capable of effectively inactivating these pathogens without compromising food quality. Traditional technologies for pathogen inactivation, including physical methods such as heating and chemical methods such as acidification, are often efficient but can cause unfavorable changes in food, significantly reducing its nutritional content and freshness of the product, and chemical-based inactivation can result in chemical residues that are hazardous to human health (15, 16).

To overcome these challenges, researchers have explored innovative inactivation techniques to inactivate foodborne viruses that go beyond traditional methods. Innovative methods for inactivating viruses in foods/water can be separated into physical (e.g., high-pressure processing [HPP]) (17, 18), natural antimicrobials (e.g., grapefruit essential oil [GFEO] and green tea extract [GTE]) (19, 20), chemical (e.g., peracetic acid (PA), ozone, and chlorine and chlorinated compounds) (8, 21), radiation-based methods (UV, pulsed light [PL], and photodynamic therapy [PDT]) (22–25), plasma and electrical discharge-based methods (e.g., cold plasma) (26), electromagnetic wave-based methods (e.g., microwave heating) (27), or their combinations (28). Compared to thermal techniques, non-thermal techniques (NTTs) and their combinations have the advantage of being able to inactivate the target microorganisms and maintain the sensory and nutritional qualities of fresh products (29).

**Fig 1:**
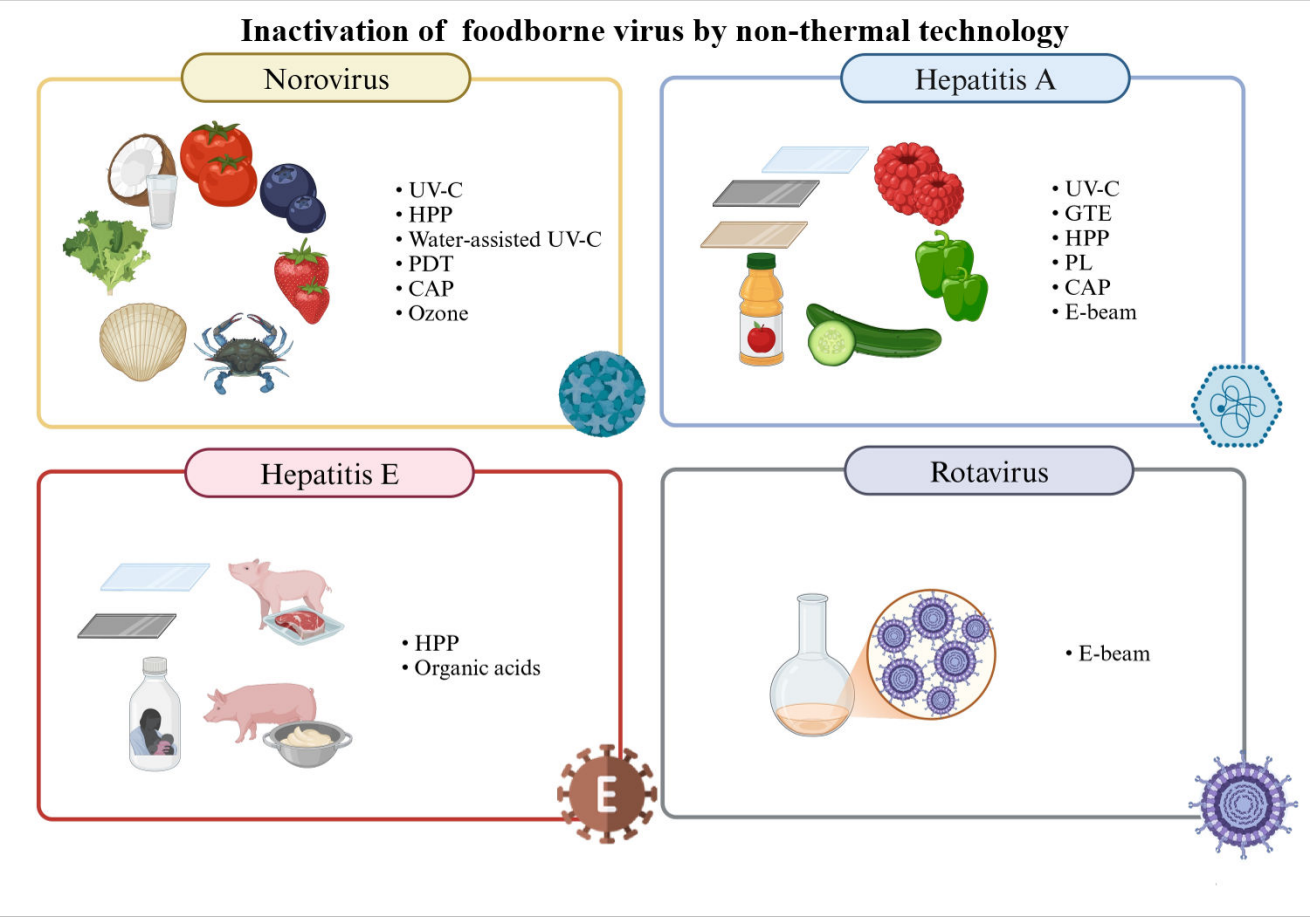
Overview of the proposed study. UV-C, ultraviolet C light; HPP, high-pressure processing; PDT, photodynamic inactivation; CAP, cold atmospheric plasma; GTE, green tea extract; PL, pulsed light; E-beam, electron-beam irradiation.

This review offers a concise overview of the main foodborne viruses and provides an update on the understanding of using non-thermal methods to inactivate these viruses and matrices where the technologies were evaluated, as represented in Fig. 1. To compose this review, a systematic literature search was conducted focusing on peer-reviewed studies published in English from 2022 onward in the PubMed, Web of Science, and Scopus databases, combining descriptors and keywords such as “viruses,” “virus surrogates,” “foodborne viruses,” “emerging technologies,” and “mechanisms of viral inactivation in food.” This resulted in 46 relevant publications. After removing duplicates and applying eligibility criteria—based on relevance, scope, and methodological depth —26 studies were selected for in-depth analysis, as it allowed us to gather current evidence on the mechanisms of inactivation of foodborne viruses, as well as to assess the perspectives and limitations of emerging technologies applied to the control of these pathogens. Websites of government or regulatory agencies such as the European Food Safety Authority, European Center for Disease Prevention and Control, Food and Drug Administration (FDA), and Centers for Disease Control and Prevention were also used to collect epidemiological information.

## FOODBORNE VIRUSES

The ingestion of food contaminated by foodborne viruses represents a serious public health problem. Outbreaks involving viruses occur when two or more people present similar symptoms, ranging from gastrointestinal symptoms to more severe conditions, which can, in some cases, lead to death, depending on the individual’s immune status. Various viruses can be involved in foodborne outbreaks; norovirus and hepatitis A virus can contaminate food, especially when consumed raw or minimally processed, representing a significant risk to consumers. In addition, hepatitis E virus and rotavirus can also cause sporadic outbreaks, especially in vulnerable populations. In low-income countries, transmission of HAV and HEV is primarily caused by contaminated water and poor sanitation. In contrast, in middle- and high-income countries, transmission is most often associated with contaminated food and outbreaks in indoor settings (HAV is associated with travel, HEV with zoonotic sources, and norovirus and rotavirus with person-to-person spread in densely populated settings such as schools and care facilities) (30–34). The main outbreaks of these viruses associated with food contamination are presented in Table 1.

**TABLE 1:** Summary of viral outbreaks associated with food contamination: epidemiological data and contextual factors

Virus	Year	Location	Illnesses	Source of contamination	Socioeconomic context	Refs
HuNoV	December 2022	Multistate outbreak (Alabama, Florida, Georgia, Louisiana, Mississippi, North Carolina, Tennessee, and Texas), USA	211 illnesses	Raw oysters potentially contaminated with norovirus were distributed to restaurants and retailers.	It affects countries of all income levels, but outbreaks are especially common in closed environments with high turnover of people, such as ships and nursing homes. Young children, the elderly, and individuals with comorbidities are among those most susceptible to developing more severe symptoms.	30, 31, 35, 36
	November 2022	Illinois, USA	227 illnesses	Improper handling of food. The person responsible for preparing the salad was symptomatic, had episodes of vomiting, and handled the ingredients without using gloves.		
	June 2022	Multistate outbreak (California, Colorado, Florida, Hawaii, Illinois, Massachusetts, Minnesota, New Jersey, Nevada, New York, Oregon, Texas, and Washington), USA	192 illnesses	Potentially contaminated raw oysters harvested in the south and central parts of Baynes Sound, British Columbia, Canada, were distributed to restaurants and retailers in 13 states.		
HAV	April 2023	Multistate outbreak (California, Hawaii, Oregon, and Washington), USA	10 cases and 4 hospitalizations	The strawberries, imported from a farm in Baja California (Mexico), were sold to a variety of retailers under multiple brand labels.	In low-income areas, HAV is more prevalent in children, but there is a risk of outbreaks in adolescents and adults due to lower levels of exposure/vaccination. In middle- and high-income countries, outbreaks occur mainly in unvaccinated adults, especially linked to travel and contaminated food (oysters and imported fruits).	32, 37
	August 2022	Multistate outbreak (Arizona, California, Minnesota, and North Dakota), USA	19 cases and 13 hospitalizations	The strawberries, imported from a farm in Baja California (Mexico), were sold to a variety of retailers under multiple brand labels.		
HEV	July to August 2022	Zhejiang Province, China	77 cases and 18 showed characteristic symptoms of HEV	The consumption of pork liver, in addition to raw fruits and vegetables, was a risk factor for the spread of the disease.	In low- and middle-income countries (such as Asia and Africa), HEV is associated with water- and food-borne outbreaks, mainly due to consumption of contaminated water and undercooked pork. Refugees and vulnerable populations are at greater risk due to poor sanitation conditions. In high-income countries, transmission is predominantly zoonotic (consumption of pork and artisanal products).	33, 38–40
	June 2022	Yantai, China	80 people and resulting in 18 acute infections	The infection was associated with the consumption of cucumber sauce, and further investigation revealed cross-contamination due to the inappropriate use of pots and pans for raw pork and cold dishes.		
RV	2017–2020, 15 RV outbreaks	Seoul, South Korea	Neonatal facilities (six in hospital neonatal wards and nine in postpartum care centers)	Not disclosed.	The infection is most prevalent in low-income areas, where limited access to sanitation and health care exacerbates transmission. The introduction of the vaccine has resulted in a significant reduction in hospitalizations and deaths. Outbreaks are most common in closed, crowded settings such as daycare centers, hospitals, and long-term care facilities.	41–44
	2018	Botswana, Africa	More than 4,500 cases	Possibly poor condition of water and sanitation systems in Botswana.		
	2017	Freiburg, Germany	32 cases	Although the complete route of transmission has not been determined, there is evidence that infection occurred through contaminated surfaces rather than through direct contact between people.		

### Human norovirus

Norovirus is a single-stranded, non-enveloped, positive-sense single-stranded RNA genome (approximately 7.7 kb in size) belonging to the *Caliciviridae* family (45). Phylogenetically, norovirus is categorized into 10 distinct genogroups (GI-GX) and two unassigned gene clusters (46), where norovirus GI and GII deserve special attention due to their global epidemic potential (47). People who have been exposed to the virus usually present symptoms such as general malaise accompanied by nausea, vomiting, watery diarrhea, occasionally fever, chills, and headache (48), between 24 and 48 hours or the first 12 hours after consumption (30).

The WHO estimates that HuNoV causes about 685 million cases per year, including 200 million in children under 5 years of age, and approximately 200,000 deaths annually, of which 50,000 occur in children, with the greatest impact in low-income countries (7). HuNoV is a major cause of non-bacterial acute gastroenteritis, affecting people all over the world, leading to costs of $60 billion worldwide due to healthcare costs and lost productivity (31). HuNoV is transmitted via the fecal-oral route, either through direct person-to-person contact or through fecal contamination of food, water, or environmental surfaces (fomites), which also play a role in transmission. Food can become contaminated with fecal material at the point of production or during food preparation (49, 50). In the United States, norovirus causes 58% of foodborne illnesses, with approximately 2,500 outbreaks reported each year (31).

Among the foods most commonly involved in HuNoV outbreaks (Table 1) are leafy greens (such as lettuce) and fresh fruits (such as berries) (51). In Europe, shellfish, mollusks, and crustaceans are the most common food implicated in outbreaks (52). Also, in the United States, all alerts issued in 2025–2024 were related to possible oyster contamination (53).

### Hepatitis A virus

HAV, belonging to the *Picornaviridae* family, is a non-enveloped virus and one of the main causes of acute viral hepatitis in the world, representing a serious risk to public health due to its wide global dissemination (54). Although sexual and blood transfusion routes have also been reported, HAV primarily spreads through the fecal-oral route, which can occur through direct contact with infected people or indirectly through the consumption of contaminated food and water (55).

WHO estimates that 159 million HAV infections occur annually worldwide, with 39,000 associated deaths (56). In 2022, 2,265 cases were reported in the United States (32). Data from the same year, 2022, reveal that 30 European Union/European Economic Area countries reported a total of 4,548 cases of hepatitis A, corresponding to a notification rate of one case per 100,000 inhabitants (57). After an incubation period that varies between 2 and 7 weeks, HAV can progress to a symptomatic phase, characterized by gastrointestinal symptoms, such as diarrhea, vomiting, jaundice, and abdominal pain (58).

Foodborne HAV outbreaks (Table 1) have been known and reported since 1956 (59). HAV outbreaks occur due to the consumption of contaminated water and foods including fresh and frozen products (such as fruits), tomatoes, onions, shellfish, leafy vegetables, and ready-to-eat foods (60, 61).

### Hepatitis E virus 

HEV is a non-enveloped positive-sense single-stranded RNA virus of the family *Hepeviridae*. Phylogenetically, HEV can be divided into eight genotypes, with HEV-1 to HEV-4 being the main ones responsible for infections in humans (62, 63). Of these, HEV-1 and HEV-2 are transmitted mainly by the fecal-oral route, with contaminated water being the main source of infection, especially in areas with lower hygiene standards (64). HEV-3 has a global distribution, and HEV-4 is primarily present in Asia (38). The HEV symptoms are usually asymptomatic or mild, including fatigue, fever, and jaundice, but in pregnant women, symptoms can be more severe or even fatal (65).

HEV causes sporadic cases and endemic outbreaks (Table 1). WHO estimates that around 20 million HEV infections occur worldwide each year, resulting in around 3.3 million symptomatic cases of HEV (33, 56). HEV is not common in the United States, where people have access to clean drinking water, so most cases involve people who have recently traveled to countries where HEV is common (39). HEV can be transmitted through various routes, such as ingestion of contaminated food and water, blood transfusions, organ transplants, and vertical transmission. Viruses present in wild animals, farm animals, and human sewage contribute to fecal-oral (zoonotic) transmission through contaminated water. Irrigation with such water can contaminate fruits and vegetables, while consumption of shellfish from affected areas and animal products, such as undercooked or raw milk and meat, increases the risk of infection (64).

### Rotavírus

RV is a non-enveloped segmented double-stranded RNA genome (16–21 kbp) virus, belonging to the *Sedoreoviridae* and *Reoviridae* family (66). RV can be divided, according to the International Committee on Taxonomy of Viruses, into 10 main groups (RVA–RVJ) (67, 68). Of these, RV A, B, and C are capable of infecting humans, with RV A the most common and responsible for most infections worldwide. Group A rotavirus has two outer capsid proteins, VP4 and VP7, which determine types P and G, respectively (69). The most prevalent G and P genotypes for human RVA strains worldwide are G1P[8], G2P[4], G3P[8], G4P[8], G9P[8], and G12P[8] (70).

RV is the most common cause of severe gastroenteritis in children under 5 years of age in developing, industrialized, and least developed countries (71, 72). The WHO estimates that rotavirus infections cause over 25 million outpatient visits, more than 2 million hospitalizations, and 528,000 deaths each year, with the highest death rates in Southern Asia and sub-Saharan Africa (34, 56). Prior to the introduction of the vaccine in 2006, an estimated 2.7 million rotavirus infections occurred each year in the United States, with a 95% exposure rate among children under age 5 (73). A summary of the outbreaks caused by RV is described in Table 1.

The fecal-oral route is considered the main route of transmission of RV, and among the main risk factors are lack of hygiene, contact with contaminated surfaces, and consumption of infected water or food (41). The incubation period for rotavirus is usually short, less than 48 hours, and the infection may be asymptomatic or symptomatic. In children, the symptoms include severe watery diarrhea, vomiting, fever, and abdominal discomfort that may last from 3 to 8 days (73, 74).

## MECHANISMS OF FOODBORNE VIRUS INACTIVATION BY NON-THERMAL TECHNOLOGIES

**Fig 2:**
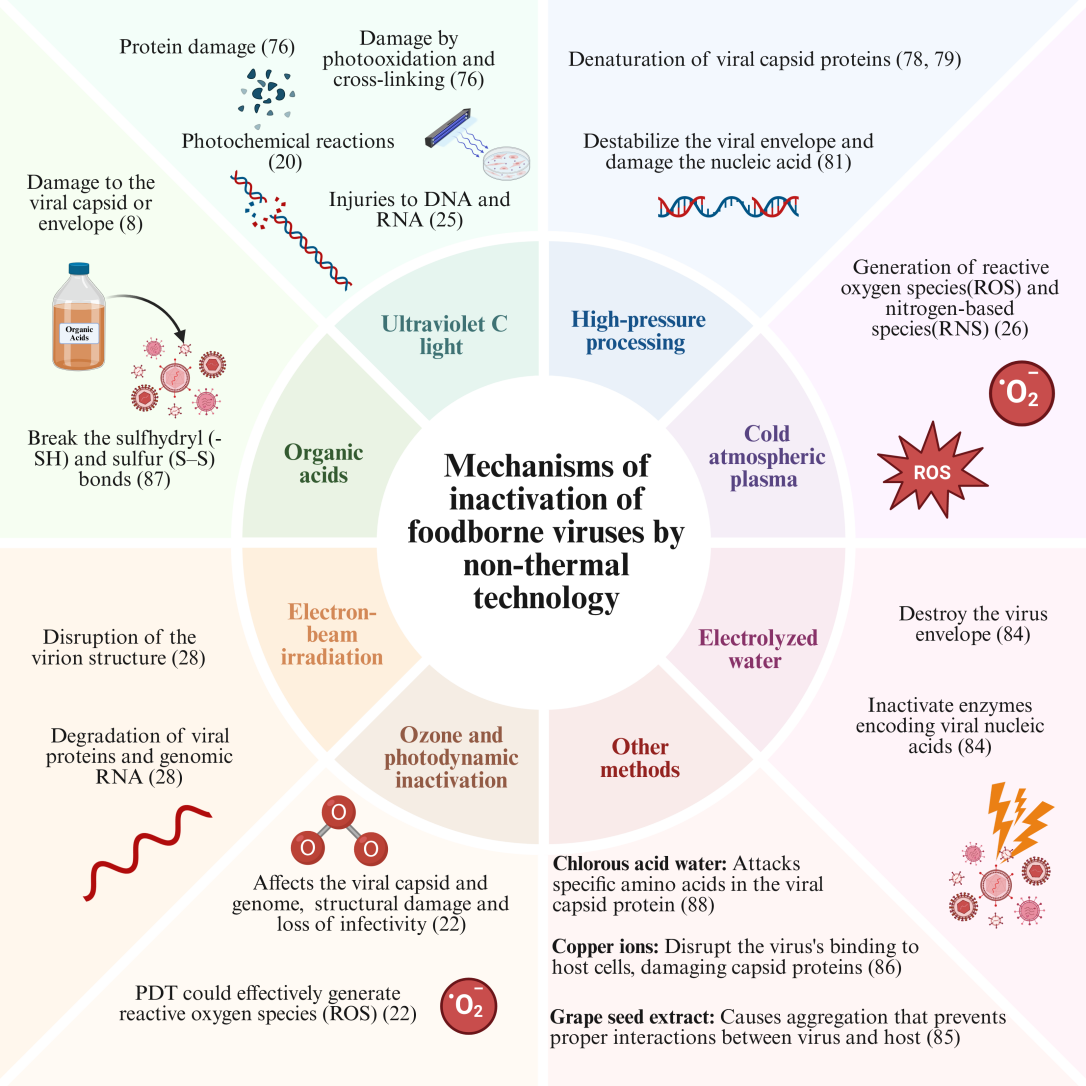
Main mechanisms proposed for viral inactivation using NTTs.

Regarding foodborne viruses and the recent outbreaks, there is a concern about developing and using emerging technologies to promote food safety. In this context, the application of NTT represents a trend. Besides presenting efficient microbial inactivation, the NTT demands less water and low energetic costs, corroborating with sustainable food production. Table 2 describes recent studies (last 3 years) on the utilization of diverse emerging technologies for foodborne viruses’ inactivation. Despite the promising findings highlighted in Table 2, there is no consensus on the proposed mechanisms of virus inactivation related to the NTT (Fig. 2), as well as standardization of the conditions and optimization of the processing. Also, the current gold-standard techniques, like PCR, can be time-consuming and expensive, limiting their widespread use in routine testing and in studies to understand the efficacy of novel technologies for food preservation (75). Thus, several studies estimate viral inactivation using viral substitutes. Studies using surrogates help to understand different key aspects of viral stability and persistence, the performance of various technological interventions, and the efficacy of inactivation agents, while considering the impact of food matrices and organic loads (49). Murine norovirus (MNV), feline calicivirus (FCV), Tulane virus (TV), and MS2 bacteriophage have been widely used as surrogates of HuNoV (20, 22, 76). However, for other foodborne viruses, the use of surrogates is not a consensus in the scientific community. Viral surrogates are selected due to the approximate physiological and morphological characteristics and behavioral characteristics (persistence, resistance, and survival) of viral pathogens. However, one of the limitations of using viral surrogates is that they may not accurately replicate the physicochemical properties, environmental persistence, or inactivation profiles of target viruses, which may result in under- or overestimation of the efficacy of the treatments being evaluated (77).

**TABLE 2:** Studies^*a*^ on the utilization of emerging technologies for inactivation of foodborne viruses: insightful findings and mechanisms

Agent	Objectives	Food/sample	Technological strategy/experimental design	Insightful findings	Mechanisms	Ref
FCV and TV	Determine the inactivation of HuNoV surrogates.	Phosphate-buffered saline and coconut water	UV-C (254 nm, 33.89 mJ/cm^2^ for up to 15 min) and UV-C LED (279 nm, 7.03 mJ/cm^2^ for 0 up to 2.5 min).	UV-C LED was more effective in inactivating HuNoV surrogates than UV-C in PBS and coconut water.	Protein and viral capsid damage induce photochemical reactions, including protein cross-linking and photooxidation, that disrupt capsid integrity and impair viral function.	76
HuNoV GII.4	Investigate the effect of HPP on the inactivation of HuNoV GII.4.	Clam *jeotgal*	HPP 100–600 MPa, 18°C, 5 min.	Inactivation was pressure-dependent. Approximately 1.5–2 log copies/L at 600 MPa treatment.	Denaturation of the viral capsid proteins, leading to the incapacitation of infection virions, inhibiting their attachment and penetration into host cells.	78
MNV and TV	Assess the effects of GFEO in combination with UV-C on vegetables.	Cherry tomatoes, lettuce, and blueberries	GFEO (0.8% [vol/vol], 0–12 min) +UV C (0–200 mJ/cm^2^).	Combined treatments achieved higher inactivation than alone treatments. The viral reduction remained effective during 7-day storage at 4°C.	Denaturation of the viral capsid proteins by GFEO, allowing UV-C penetration into pathogen particles and subsequent inactivation by photochemical reactions and cross-linking.	20
HAV and FCV	Compare the effects of UV-C LED to UV-C against HAV and FCV.	Stainless steel, ceramic, and glass surfaces	UV-C LED (279 nm) and UV-C (254 nm) 0–49.2 mJ/cm^2^ for up to 3.75 min for HAV, and 0–24.6 mJ/cm^2^ for FCV.	UV-C LED requires higher doses for inactivation of HAV and FCV regardless of surface type.	Injuries to DNA and RNA due to photochemical changes, cross-linking, and photooxidative damage, inhibiting DNA replication.	25
HuNoV	Evaluate the viral capsid integrity of HuNoV GI and GII.	Oysters	HPP 300–600 MPa, 15°C–20°C, 5 min	Capsid integrity was more influenced by at 15°C under 450 MPa, showing temperature dependency. Norovirus GI.3 required 600 MPa at 20°C for effective reduction, whereas GII.4 was inactivated at 300 MPa.	Denaturation of viral capsid proteins, leading to the incapacitation of infection virions, blocking their attachment and viability.	79
HEV and Coronavirus	Compare the efficacy of HPP with the HoP on virus-infected human milk.	Human milk	HPP 350 MPa (4×), 38°C and 600 MPa (1×), 20°C.	Milk does not protect HEV from HPP inactivation. HPP does not completely inactivate surrogate models, indicating that these treatments cannot ensure full viral safety.	Not detailed.	80
HEV	Evaluate the effects of HPP in the inactivation of HEV.	Prosciutto	HPP 300–600 MPa, 9 min.	HEV counting decreased when using HPP up to 400 MPa; however, treatment at 500 MPa did not differ from control.	HPP can destabilize the viral envelope and damage the nucleic acid of HEV, preventing replication.	81
HuNoV	Assess the efficiency of HPP in reducing HuNoV.	Preserved raw crab	HPP 200–600 MPa, 6°C, 5 min	Despite the viral inactivation (2.34 log at 600 MPa), raw crab demonstrated significant changes in instrumental color, pH, and sensory properties.	HPP may cause viral protein damage.	82
MNV	Examine the inactivation ofMNV-1 on whole and fresh-cut strawberries.	Whole and fresh-cut strawberries	Water-assisted UV-C and PA Four different disinfection treatments: water (H_2_O), chlorine (NaClO) 200 mg/L; water-assisted UV-C and PA 40 mg/L.	MNV-1 reduction after disinfection ranged from 1.3 to 1.7 log, but the virus persisted on strawberries stored at 4°C or 10°C for 7 days, even after treatment.	UV-C and PA combination induce the photolysis of PA, with disruption in the O–O bond of the PA molecule and the formation of hydroxyl radicals, which are harmful to the microbial DNA.	21
HAV	Compare the efficacy of HPP with the HoP on virus-infected human milk.	Human milk	HPP 350–600 MPa, *<*10°C, 8–10 min.	HPP presented a higher inactivation (>4 log PFU/mL) than HoP (3.1 log PFU/mL) in human milk. Macronutrients (protein, fat, and carbohydrate) of milk were not affected by the treatments.	Not described.	18
MNV and HAV	Evaluate the effectiveness of CAP in inactivating MNV and HAV on fruit	Raspberries	CAP Positive corona discharge fed by synthetic air, 1–10 min, 25 W.	Applying CAP allows the inactivation of 4 log for MNV and HAV after 5 and 10 min treatment, respectively. The authors described a time-dependent structural deformation of the viral capsid upon CAP treatment.	During the treatment, reactive oxygen species (ROS) and nitrogen-based species are generated, which in combination with the UV radiation efficiently inactivate the virus.	26
HuNoV, MNV, and TV	Assess the efficiency of HPP in reducing HuNoV.	Strawberry puree	HPP 350–450 MPa up to 5 min.	TV was more resistant than MNV to HPP. At 5 min and 450, HPP inactivated the HuNoV GII.4 and surrogates in PBS and strawberry puree, though the food matrix had a baroprotective effect.	Not described.	17
MNV and HAV	Assess the effects of the treatment on the visual characteristics of various frozen fruits and on the infectivity of MNV-1 and HAV.	Frozen fruits	PL 16 pulses, 11.52 J/cm^2^, distance ranging from 7 to 7.5 cm.	Antiviral effect depends on the type of fruit. PL caused a rise in temperature on the product surface; however, no visible physical changes were observed.	Not described.	23
HuNoV, MNV, and HAV	Investigate different HPP and GTE treatments, individually and in combination, and define the synergistic antiviral effect of these two technologies.	Beverages (apple juice and horchata)	HPP + GTE 300–500 MPa, 5 min + GTE.	HPP treatment completely inhibits HuNoV GII.4 infectivity when applied at 500 MPa alone and at 400 MPa combined with aged-GTE.	HPP-induced capsid damage permits aged-GTE entry, allowing interference with viral RNA. Additionally, GTE contributes to inactivation through its oxidizing activity and ability to block histo-blood group antigen binding.	19
MS2 bacteriophage	Assess the efficacy of ozone and PDT against the Norovirus surrogate.	Brazilian berries (black mulberry and pitanga) and surfaces (glass and stainless steel)	Ozone and PDT-curcumin Ozone: bubble diffusion in water, 6.25 ppm, 30 s to 20 min. PDT-curcumin: LED emission, 33.6 mW/cm^2^ irradiance, 16.1–24.2 J/cm^2^ light dose, 8–12 min.	Ozone was more effective at inactivating MS2 on surfaces, whereas PDT-curcumin showed greater efficacy on Brazilian berries, both achieving reductions of up to 4.8 log PFU/cm^2^.	Ozone causes oxidative damage to the viral capsid and genome, disrupting structure and infectivity, while PDT generates ROS that inactivate viruses by damaging their particles.	22
MNV and TV	Evaluate the potential of subzero temperature to enhance the inactivation of norovirus surrogates.	Bay oysters	HPP-low temperature 200–300 MPa, 5 min.	MNV presented a higher inactivation (>6.1 log reduction) than TV (>4.0 log reduction) at 300 MPa. The state of the samples (thawed vs frozen) affects the effectiveness of HPP.	Not described.	83
HAV	Investigate the synergistic effects of chemical and physical sequential treatment for HAV inactivation.	Bell pepper and cucumber	NaOCl/ClO_2_ + e-beam NaOCl (50–500 ppm), ClO_2_ (10–250 ppm) and e-beam (1–5 kGy).	500 ppm NaOCl + 3 kGy on bell pepper and 150 ppm NaOCl + 1 kGy on cucumber provided maximum synergistic effects. 50 ppm ClO_2_ + 5 kGy on bell pepper and 10 ppm ClO_2_ + 5 kGy on cucumber inactivated HAV more efficiently.	NaOCl and ClO_2_ damage viral nucleic acids and proteins through oxidation, while E-beam disrupts virion structure by degrading proteins and RNA.	28
MNV and TV	Assess the efficacy of PL against HuNoV surrogates.	Bacterial biofilms	PL 16 pulses at intervals of 1,186 ms (total energy input to 11.52 J/cm^2^).	Treatment significantly reduced the viable bacterial cells in all single-species biofilms. TV was significantly more resistant to PL than MNV-1. The composition of the biofilm matrix may contribute to virus resistance.	Viral reductions are associated with treatment-induced capsid impairment but did not detail the mechanism.	24
MS2 bacteriophage	Estimate the effects of microwave heating in inactivation of the norovirus surrogate.	Frozen strawberries	Microwave heating 30%–100%, 15–300 s	The lowest power level showed the slowest MS2 inactivation. Heating at 100% power showed the most rapid bacteriophage inactivation, which was not detected after 90 s of treatment.	Not described.	27
HuNoV	Investigate the effects of the combination of SAEW and UV-C LED irradiation against HuNoV on surface.	Stainless-steel surface	SAEW and UV-C LED SAEW droplets (60–180 μL), available chlorine concentration (4–30 ppm), and UV-C LED dose (1.08–3.24 mJ/cm^2^), intensities of 18 μW/cm^2^, 60–180 s.	The combined treatment was efficient in inactivating HuNoVs. The conditions of 180 µL SAEW, 30 ppm of chlorine concentration, and 2 mJ/cm^2^ UV-C LED dose resulted in the highest HuNoV inactivation (3.21 log_10_ genome copies).	SAEW disrupts viral envelopes, damages surface proteins, inactivates nucleic acids, and destroys RNA. UV-C causes oxidative damage to capsid proteins and, in some cases, the viral genome.	84
HRV	Evaluate the effectiveness of electron beam irradiation as a method of inactivating pathogens.	Viral solution	E-beam 2–15 kGy.	Complete inactivation of HRV was achieved at 15 kGy.	Not described.	9
TV	To examine Grape seed extract (GSE)-induced inactivation of TV.	Viral solution	GSE 84–1,694 mg/mL, 10–120 s.	TV inactivation was dependent on GSE concentration and incubation duration. The antiviral efficacy of GSE depends on initial virus titers.	GSE causes TV aggregation that prevents proper virus-host interactions or virus entry into host cells.	85
TV	Evaluate the effects of copper ions on virus inactivation and estimate the resulting loss of HuNoV infectivity and capsid integrity.	Viral solution	Copper ions CuBr_2_ solutions (0.01–1 mM), 30 min. CuBr_2_ (0.001–1 mM) in combination with 10× excess ascorbate for 30 min.	Cu^+^ demonstrated significantly greater antiviral efficacy than Cu^2+^. Low concentrations of Cu^+^ could achieve significant reductions in viral infectivity.	Cu(I) ions can disrupt virus binding to host cells by damaging capsid proteins, which prevents the virus from binding to cellular receptors.	86
HEV	Examine the effectiveness of organic acids in the inactivation of HEV on food contact surfaces and pork products.	Plastic, stainless steel, and pork pâté surfaces	Citric acid and acetic acid 1%–5%, 10 min, 22°C, 30%–40% RH.	The greatest reductions in HEV viral infectivity were observed on plastic and stainless steel compared to pork pâté.	The damage to the viral capsid or envelope is considered the main reason for viral inactivation by organic acids.	8
MS2 bacteriophage	Evaluate the efficiency of preservation technologies in the inactivation of MS2 in fruit pulps.	Raspberry and pitanga pulps	Ascorbic acid (1 mg/mL), citric acid (0.3 mg/mL), sodium metabisulfite (5 mg/mL), and sodium benzoate (5 mg/mL), 4°C, 24 h.	The addition of organic acids, sodium benzoate, or sodium metabisulfite had little effect on the inactivation of MS2.	Some organic acids may disrupt viral structures by breaking sulfhydryl (-SH) and disulfide (S–S) bonds in capsids.	87
HuNoV	Investigate the capability of CA vs NaClO to inactivate HuNoV.	Viral solution	CA + NaClO CA 200 ppm, 30 min. NaClO 200–500 ppm, 30 min	CA (200 ppm) and NaClO (200 ppm/500 ppm) provided >3.0 log10 reduction in the HuNoV RNA within 30 min. The effectiveness of CA was maintained even under organic-matter-rich conditions.	CA damages HuNoV by attacking specific amino acids in the viral capsid protein.	88

^*a*^ From 2022 to date. HRV: human rotavirus; CA: chlorous acid water; CAP: cold atmospheric plasma; E-beam: electron-beam irradiation; HoP: holder pasteurization method; UV-C: UV C light; LED: light-emission diode; UV-LED: UV LED light; SAEW: slightly acidic electrolyzed water.

### High-pressure processing

The principle of the utilization of HPP is based on the generation of mechanical pressure by a piston in a liquid medium containing the packaged food, so the pressure is uniformly distributed (89). According to Wang et al. (90), HPP can be applied on different food matrices, mainly the sterilization of fruit, vegetables, and dairy products, flavor improvement of alcoholic beverages, and tenderization of meat and fish products. HPP alters the proteins’ secondary, tertiary, and quaternary structures by promoting conformational changes, inactivation of enzymes, disruption of cell membranes, and shifting the internal arrangement (89). The bacterial inactivation mechanism of HPP includes the effects against the cell membrane, causing disruption and alteration of membrane permeability, morphological changes, and denaturation of enzymes and proteins, reducing metabolic activity and ribosomal synthesis (91). However, the mechanisms behind viral inactivation remain unclear. Jeon et al. (78) described HuNoV inactivation associated with the denaturation of the viral capsid proteins, which are essential during the entrance and exit of the virions from the host cell, conferring protection. In this context, the increase in the employed pressure (100–600 MPa) was a significant factor contributing to a higher inactivation. Similar findings were depicted by Roy et al. (82), which reported a high HuNoV reduction at 600 MPa. In agreement, Rachmadi et al. (79) reported pressure-dependent behavior for HuNoV inactivation, suggesting the denaturation of the viral capsid proteins as the main mechanism of viral reduction. Moreover, the authors hypothesized that low temperatures, around 15°C, had a beneficial effect when combined with HPP. In contrast, Lee et al. (81) suggested that HPP can reduce the stability of the HEV envelope and promote damage in the nucleic acids because of the disruption of the envelope and release of RNA. Despite the promising findings, none of these authors applied different conditions of pressure and time in their studies. Therefore, it is difficult to establish optimal conditions for the use of HPP. Besides, most of the studies on HPP viral inactivation focused on solid food matrices, including clams, oysters, crabs, and ham, and data about liquid food are still scarce (Table 2). However, it has been proposed that HPP could expose active regions of milk fat globule membranes, thereby facilitating viral inactivation in products such as milk (92). Moreover, the combined use of HPP and GTE was recently assessed by Falcó et al. (19) in beverages. HPP was responsible for capsid viral damage, allowing the entrance of GTE and affecting the viral replication at the RNA level. Therefore, considering the previous findings, it can be stated that viral inactivation induced by HPP is caused by (i) denaturation of the viral capsid proteins, (ii) instability and disruption of the envelope, and (iii) release and damage to the nucleic acids, which block the viral replication.

### Light-emission technologies

UV C light (UV-C) is an NTT generally employed to promote the decontamination of surfaces and is approved for use by the FDA (93). According to Tchonkouang et al. (94), the UV-C radiation technology presents affordable costs to the food industry, requiring a low initial investment. It is described as cost-saving compared to heat pasteurization and to other non-thermal technologies, such as HPP. The range of utilization for microbial reduction is around 254 nm since, at this wavelength, the nucleic acids of the bacterium are recognized as strong light absorbers, improving efficiency (95). The UV-C light-emission diode (LED) is a derivative of UV-C in which the main advantage is the use of LED lamps instead of mercury lamps, reducing economic costs and decreasing potential risks to the consumer and environment (96). Both technologies can act by forming cytosine and thymine crosslinks via hydrogen bonds, resulting in pyrimidine dimers that hinder metabolic synthesis (95). Also, UV technologies induce the formation of reactive oxygen species (ROS), causing oxidative stress reactions on the target (97). In the present review, we identified five studies that proposed the mechanism of viral inactivation by UV-based technologies (Table 2). Corson et al. (76) and Polen et al. (25) attributed the inactivation of feline calicivirus, Tulane virus, and HAV to photochemical damage and protein crosslinks induced by the UV treatments. However, both studies stated that 279 nm UV-C LED presents better effectiveness than the conventional 254 nm UV-C. The hypothesis indicated by the authors is that at 279 nm wavelength (UV-C LED), there is a higher light absorption by the viral capsid proteins in comparison with 254 nm (UV-C) (21). Regarding the combined effects of UV-C and biological and chemical compounds, Liao et al. (20) reported that GFEO could induce the denaturation of capsid proteins of HuNoV surrogates, facilitating the photochemical inactivation. The GFEO is mainly composed of limonene, a bioactive compound with recognized antiviral activity (98). On the other hand, Ortiz‐Solà et al. (21) pointed out that the use of PA potentialized the effects of UV-C since UV-C induces PA photolysis and the formation of hydroxyl radicals, magnifying the ROS generation and the damage to nucleic acids. In addition, the influences of slightly acidic electrolyzed water (SAEW) combined with UV-C LED against HuNoV were optimized by Song et al. (84), considering the amount and concentration of SAEW and the dose of UV-C LED. All the factors assessed by the authors were significant for the reduction of viral charge. In this context, SAEW destroys the viral envelope, damaging proteins and nucleic acids. Therefore, it is important to note that, considering the optimization study and the significance of the factors, it is possible to interchange the values of the factors and use different combinations to achieve similar reduction levels.

PL, also known as pulsed UV light, is another light-based technology with proven antimicrobial activity. It is the application of intense light in the form of short pulses against the target, being similar to sunlight, covering a wide wavelength range (200–1,100 nm) (99). The lethal effects are mainly photochemical, like the UV at 254 nm; however, there are also photothermal and photophysical influences on the matrix (100). There are few studies exploring the mechanisms of PL against foodborne pathogens (Table 2). Péloquin et al. (24) suggested that the interaction between viruses, such as Tulane virus and HuNoV, with bacterial biofilms could increase the viral resistance against PL treatments, representing a limitation for the use of this technology. In contrast, Kim et al. (23) reported that the application of PL on fruits is beneficial, aiming for the inactivation of HAV, but suggests that PL should be employed in combination with other barriers to improve the decontamination efficiency by synergistic effects. Despite the promising scenario of light technologies, there is no consensus about which is the best technology or the viral mechanisms of resistance against them. Therefore, further research should focus on performing a reliable comparison of light technologies, considering (i) the mechanisms of viral resistance, (ii) the energy input (dose), and (iii) the characteristics of the food material, such as thickness and physical state.

### Other emerging technologies

Despite HPP and UV-C being the main NTT applied for viral inactivation in the last 3 years, other emerging technologies have been evaluated, including cold atmospheric plasma (CAP), ozone, electronic beam (e-beam), and microwave (Table 2). CAP’s inactivation mechanism is similar to UV-C regarding the induced oxidative stress. In this sense, Velebit et al. (26) treated raspberries with CAP at different times (1–10 min) and reported around 4 log reductions of murine norovirus and HAV. The viral reduction was attributed to generating ROS and reactive nitrogen species, such as hydroxyl radicals, superoxide anion, nitric oxide, etc., leading to injuries in the genetic material. A similar mechanism was reported by De Souza Grilo et al. (22), assessing the inactivation of norovirus surrogates by ozone and PDT. PDT acts as a photoinactivation system, in which an exogenous photosensitizer produces ROS after exposure to blue light (101). According to the authors, ozone causes chemical injuries in the virus capsid and genome by photooxidation of lipids and proteins, while PDT generates intracellular ROS, attacking intracellular structures (22). Lastly, Son et al. (28) evaluated the effects of sequential application of NaOCl, ClO_2_, and e-beam against HAV. NaOCl induces damage in nucleic acids and capsid proteins after dissociation into HOCl^−^, while ClO_2_ can also oxidize tryptophan and tyrosine, increasing the antiviral activity. The authors reported a synergistic effect when combining NaOCl or ClO_2_ with e-beam.

In general, the NTT discussed above, i.e., HPP, UV-C, PL, CAP, and ozone, is well established in the literature for bacterial inactivation in food systems but remains scarce in the studies focusing on viral load reduction. However, despite the promising findings in the last years, as well as a positive appeal regarding energy savings and environmental impact, most of these technologies still present challenges for industrial application, such as (i) cost-effectiveness, (ii) induction of undesirable physicochemical changes, e.g., protein and lipid oxidation and, consequently, quality losses, and (iii) resistance by the consumers to accept novel products processed by NTT (102, 103). Therefore, its adoption at the industrial level must consider a risk-benefit analysis, not only the viral inactivation efficiency.

### Bioactive and chemical compounds

Besides the NTT previously discussed, some studies investigated the use of biological and chemical molecules as alternatives to promote viral inactivation, including fruit extracts, organic acids, chlorine compounds, and copper. Oh et al. (85) employed commercial grape seed extract (GSE) and obtained positive results in inactivating the Tulane virus. The main hypothesis discussed by the authors is that GSE can directly bind to the virus and induce viral aggregation, blocking further interaction with the host cells. The binding between GSE and the vial particle could be mediated by the polyphenol’s contact with the capsid protein at different locations (85). In this context, bioactive compounds seem to be a suitable alternative in view of traditional technologies and NTT, since, besides the antimicrobial activity, they also present antioxidant properties, maintaining the oxidative stability of food and extending shelf life (97). In the case of organic acids, McLeod et al. (8) suggested that damage to the viral capsid is the main cause of the reduction of HEV. The use of acetic acid induced a greater reduction when compared with citric acid; however, there was no difference between increasing concentrations (1%–5%). Besides the pH alteration induced by organic acids, there is an accumulation of intracellular ions that corroborate with the inactivation. Moreover, some organic acids, such as citric acid and ascorbic acid, can break sulfhydryl and sulfur bonds in the capsid (87). Notwithstanding, chlorous acid water (CA) at 200 ppm has been proven to have effects in inactivating HuNoV (88). CA contains HClO_2_ as a component, which does not lose the antiviral activity in the presence of organic matter, different from conventional chlorine-based sanitizers, such as NaClO (88). Finally, copper ion solutions have been recently investigated against HuNoV. Mertens et al. (86) reported that monovalent copper ions at low concentrations (0.1 mM) induce capsid protein damage, reducing the total count to approximately 4 logs and preventing the binding to cell receptors; however, this kind of inactivation seems to be strain-dependent since GII.4 exhibits greater resistance than GI.7.

Therefore, as presented here, different NTT, such as HPP and UV-C, and bioactive compounds have been suggested as alternative methods for reducing viral charge in food and surfaces. However, these emerging technologies still present challenges and gaps needing further investigation to ensure efficiency and application at the industrial level, such as the need for optimization and the lack of standardization.

## CHALLENGES AND PERSPECTIVES

According to the recent findings described in this review, the application of NTT, despite its hopeful potential for microbial load reduction, still presents challenges regarding the inactivation of foodborne viruses. One of the main challenges is optimizing the parameters of each technology, such as pressure (HPP), dose (UV-C), and time, among others. The optimization of NTT processing could ensure not only reaching the maximum target pathogen inactivation, thereby increasing food safety, but also the minimization of physicochemical and sensorial changes, thus avoiding quality losses and contributing to a reduction in processing time, leading to energy savings. As reported by Bernardo and Conte-Junior (104), there is a wide range of studies on the modeling and optimization of the NTT applied to bacterial inactivation. Statistical methods, such as the Box-Behnken design (BBD) and central composite rotatable design, are widely used as approaches in this kind of study. In this context, complementary methodologies, like the desirability profile, are associated with optimization design to achieve a complete inactivation of the target (104). Among the studies included in our review, Song et al. (84) employed a BBD for modeling and optimization of HuNoV inactivation using SAEW combined with UV-C LED. The authors reached 3.21 log inactivation at optimal conditions (180 µL SAEW, 30 ppm SAEW, and 2 mJ/cm^2^ UV-C LED).

On the other hand, despite the possibility of applying optimization approaches to enhance the efficiency of the NTT, another challenge related to the non-thermal processing is the undesirable oxidative stress generated in the matrices, such as lipid and protein oxidation, leading to physicochemical changes in quality and sensory properties (105). In this context, a few authors have investigated quality alterations in food products after non-thermal processing for viral reduction (Table 2). Regarding animal products, Jeon et al. (78) reported progressive pressure-dependent instrumental and sensory changes in clams subjected to HPP. The effect of the pressure on the midgut gland justified the darkening of the samples. On the other hand, Roy et al. (82) reported higher values of lightness in crabs treated with HPP, which could be attributed to the denaturation and coagulation of protein (106). Also, in the case of prosciutto, the samples treated with HPP exhibited lower hardness than the control (81), suggesting that beyond the viral inactivation, this NTT can contribute to the tenderization due to protein denaturation and aggregation (89). Concerning fruits, the use of CAP was suggested to cause the darkening of the samples depending on the exposition time, resulting in water losses and melting of the epicuticular surface (26). Another key challenge regarding viral inactivation is the specificity of the food matrix, as reported by De Souza Grilo et al. (87). The authors pointed out that the greater reduction of MS2 bacteriophage titer in raspberry than in pitanga fruit, subjected to sodium hypochlorite, is due to the presence of bristles on the surface of the raspberry, which exposes the attached viral particles and facilitates their inactivation. Also, the use of copper ions, as suggested by Mertens et al. (86), could induce toxic reactions on human organisms when consumed at high concentrations, including several types of cancers (107).

In this regard, other strategies can be adopted to achieve viral inactivation without causing quality and sensory alterations, such as essential oils (EO). The efficiency of EO on bacterial reduction is well-known and can be increased by the development of nanoemulsions, as reviewed by Da Silva et al. (108), which improve their delivery and solubility, as well as the bioavailability of the nanocide compounds (108). Therefore, studies on the development of EO and nanoemulsions for the inactivation of foodborne viruses represent promising alternatives. Among the studies discussed in this review, only Liao et al. (20) described the application of EOs as a food safety strategy. The authors related that GFEO can be used to inactivate murine norovirus without significant alterations in the instrumental color, texture, or sensory properties. However, the mechanism of inactivation is not well understood and could be related to the presence of limonene, which binds and damages the viral capsid (20).

## CONCLUSIONS

Foodborne viruses are clearly unable to multiply in the food matrix, but their ability to survive and persist in food, food surfaces, and water is well documented, leading to food contamination, which is a relevant problem for food safety. In this context, the inactivation of foodborne viruses is an essential measure. However, traditional methods are becoming obsolete, requiring the adoption of novel methods, such as those presented in this review. We emphasize that, despite significant advances in the area, further research is needed to optimize existing methods, expand their application to different matrices, and explore the combination of different approaches to enhance viral inactivation while reducing technical parameters, such as the treatment time. Current literature does not describe any method capable of completely inactivating viruses, since different types of viruses exhibit different sensitivities in relation to the inactivation processes used. Therefore, novel inactivation methods developed must employ specific and effective approaches, adapted to the characteristics of each virus.
